# Supplementary material for: Clients satisfaction at primary healthcare facilities and its association with implementation of client service charter in Tanzania
Source: PLoS One. 2022 Aug 15;17(8):e0272321. doi: 10.1371/journal.pone.0272321 (PMC9377608; doi:10.1371/journal.pone.0272321)
Supplement: Attachment — Submitted filename: Response to reviewers.docx [file pone.0272321.s001.docx]

PONE-D-21-39135

Clients satisfaction at primary health-care facilities and its association to status of client service charter implementation in Tanzania

PLOS ONE

Dear editor, we humbly submit the responses to comments raised by the reviewers including you. Thank you again for giving us the opportunity.

Additional Journal requirements:

1. Please ensure that your manuscript meets PLOS ONE's style requirements, including those for file naming.

As per guidelines, all headings for major sections in the manuscript have been increased in font size from 16 to 18.

As per guidelines, all headings for sub-sections of major sections in the manuscript have been increased in font size from 14 to 16.

As per guidelines, all headings for level 3 in the manuscript have been increased in font size from 12 to 14.

An extension “.tif” has been added to each figure name.

Figures are now cited as “Fig 1”, “Fig 2” and “Fig 3” instead of “Figure 1”, “Figure 2” and “Figure 3” respectively.

Figures are now captioned as “Fig. 1”, “Fig. 2” and “Fig. 3” instead of “Figure 1”, “Figure 2” and “Figure 3” respectively.

Figure titles are now bolded but not italicized.

All titles have been written in sentence case

2. Thank you for stating the following in the Competing Interests section: 
[I have read the journal's policy and the authors of this manuscript have the following competing interests: During the time of baseline and reassessment as well as during write up of the study – JH, EE and TY were with the Health Quality Assurance Divison (now called Health Quality Assurance Unit) and were responsible for the implementation of SRA and QIPs folllow-up.]

Please confirm that this does not alter your adherence to all PLOS ONE policies on sharing data and materials, by including the following statement: "This does not alter our adherence to  PLOS ONE policies on sharing data and materials.” (as detailed online in our guide for authors http://journals.plos.org/plosone/s/competing-interests).  If there are restrictions on sharing of data and/or materials, please state these. Please note that we cannot proceed with consideration of your article until this information has been declared.

Please include your updated Competing Interests statement in your cover letter; we will change the online submission form on your behalf.

Thank you. The statement is included in the cover letter as instructed.

3. We note that Figure 3 in your submission contain map images which may be copyrighted. All PLOS content is published under the Creative Commons Attribution License (CC BY 4.0), which means that the manuscript, images, and Supporting Information files will be freely available online, and any third party is permitted to access, download, copy, distribute, and use these materials in any way, even commercially, with proper attribution. For these reasons, we cannot publish previously copyrighted maps or satellite images created using proprietary data, such as Google software (Google Maps, Street View, and Earth). For more information, see our copyright guidelines: http://journals.plos.org/plosone/s/licenses-and-copyright.
 
We require you to either (1) present written permission from the copyright holder to publish these figures specifically under the CC BY 4.0 license, or (2) remove the figures from your submission:
 
a) You may seek permission from the original copyright holder of Figure 3 to publish the content specifically under the CC BY 4.0 license.  
 
We recommend that you contact the original copyright holder with the Content Permission Form (http://journals.plos.org/plosone/s/file?id=7c09/content-permission-form.pdf) and the following text:
“I request permission for the open-access journal PLOS ONE to publish XXX under the Creative Commons Attribution License (CCAL) CC BY 4.0 (http://creativecommons.org/licenses/by/4.0/). Please be aware that this license allows unrestricted use and distribution, even commercially, by third parties. Please reply and provide explicit written permission to publish XXX under a CC BY license and complete the attached form.”
 
Please upload the completed Content Permission Form or other proof of granted permissions as an "Other" file with your submission.
 
In the figure caption of the copyrighted figure, please include the following text: “Reprinted from [ref] under a CC BY license, with permission from [name of publisher], original copyright [original copyright year].”
 
b) If you are unable to obtain permission from the original copyright holder to publish these figures under the CC BY 4.0 license or if the copyright holder’s requirements are incompatible with the CC BY 4.0 license, please either i) remove the figure or ii) supply a replacement figure that complies with the CC BY 4.0 license. Please check copyright information on all replacement figures and update the figure caption with source information. If applicable, please specify in the figure caption text when a figure is similar but not identical to the original image and is therefore for illustrative purposes only.
The following resources for replacing copyrighted map figures may be helpful:
 
USGS National Map Viewer (public domain): http://viewer.nationalmap.gov/viewer/
The Gateway to Astronaut Photography of Earth (public domain): http://eol.jsc.nasa.gov/sseop/clickmap/
Maps at the CIA (public domain): https://www.cia.gov/library/publications/the-world-factbook/index.html and https://www.cia.gov/library/publications/cia-maps-publications/index.html
NASA Earth Observatory (public domain): http://earthobservatory.nasa.gov/
Landsat: http://landsat.visibleearth.nasa.gov/
USGS EROS (Earth Resources Observatory and Science (EROS) Center) (public domain): http://eros.usgs.gov/#
Natural Earth (public domain): http://www.naturalearthdata.com/

Figure 3 is our own construction and was not accessed from other sources than us, authors.

GIS software version 2.8.6 was used to display the spatial distribution of the Proportion of Tanzanian Primary Health Facilities whose clients were satisfied with services. The shapes file that was used to construct the map is unrestrictedly available at the National Bureau of Statistic’s website: <https://www.nbs.go.tz/index.php/en/census-surveys/gis>

**Reviewers' comments:

Reviewer's Responses to Questions**

**Reviewer #1**: Manuscript should be revised by a native English speaker. Can you tell some reasons for facilities not being available in the data base? How was random selection of persons to interview organized? List of references could be shortened.
Thanks for your efforts!

It is not true that some facilities were not available in the database, the true statement should have been “some facilities had missing/incomplete data”. We targeted all facilities that were assessed through Star Rating Assessment (SRA). However , some had missing data during data extraction and were excluded from the analysis. The methodology section has added this statement to clarify the previous statement.

How was random selection of persons to interview organized?

We have added a description (in data sources-dependent variable subsection) on how clients for exit interviews were randomly selected in the current version.

“If there was more than one client exiting the facility, the first client to be interviewed was randomly selected by using a lottery system. In this system, each client was assigned a number and thereafter each number was written on each piece of paper. All papers were shuffled well and one paper was picked randomly to obtain the first interviewee. After returning from an interview, the data collector selected the next patient entering the consultation room until the targeted sample size was achieved.” The reference for the methodology is provided as well.

List of references could be shortened.

We have refined some references

**Reviewer #2: DEAR Author,**

The study’s topic, “Clients satisfaction at primary healthcare facilities and its association to the status of client service charter implementation in Tanzania” it’s interesting, but some sections should be clarified.
-The methodology is unclear, such measure tool or scare lack references support, and lack reliability and validity, and “Explain the purpose of this 5-minute interview to the client…” how to mention the Questionnaire could be measured.

We have added some references to support our methodologies. Details on how the exit interview was conducted have been added as well.

-In addition, the study’s satisfaction score points present dichotomy, only yes or no, why result could be presented percentage.

“Yes” and “No” were the possible values for each question. To obtain the proportion (in percentage) of the facilities scoring yes for each question; the number of facilities scoring “yes” was the numerator and the total number of facilities included in the analysis was the denominator.

-Result section, Figure 2: the satisfaction with services data why lower more than 80%, suggest author address more.

Yes, 72.2% of PHC facilities had their clients satisfied with services provided on a day of assessment. This finding is below the target value of 80%.

-“Table 4. Predictors of clients’ satisfaction at healthcare facilities during SRA of 2017/18.” incorrectly presented, samples could not match the study’s text, please check.
Some corrections have been made.

-For more contributions, suggest authors should clarify each clients satisfaction item and analyze the samples which clients satisfaction item is more meaningful to be improved. Such as patient characteristics or facilities health workers’ quality care.
-The discussion section should be reworded based on the incorrect analysis result.
Thank for the comment. We have improved some of the paragraphs in this section.

-Page 18, “Second, the coronavirus disease of 2019 (COVID-19) has exacerbated the already weak health systems in low- and middle- income countries threatening to disrupt services…” it does not include the study range, so I am confused because page 11 authors presented “….By 2017 Tanzania had 7,289 PHC facilities that were all involved in SRA assessment of the year 2017/2018.”

It is true the results of this study were obtained before the onset of the covid-19 pandemic. However, given the current situation in Tanzanian facilities and what we have learned after the covid-19 outbreak, we thought it was good to advise the government and stakeholders what could be the implication of the past findings in the current situation. And therefore they need to put in place robust systems to protect health care consumers from merging and re-emerging outbreaks/harm in the future.

Thank you for your effort.

**Reviewer #3: Introduction**

Those who are not familiar with primary healthcare do not really know what the Astana Declaration is. Please elaborate a bit more in detail.

Details on the declaration has been added.

Patient satisfaction in primary care is a controversial outcome as international studies have shown ceiling effects. Please discuss.

A ceiling effect is said to occur when a high proportion of subjects in a study have maximum scores on the observed variable. We have discussed this as one of the limitations to our findings in manuscript text. The reference has been added as well.

On page 4 it is stated „This paper discusses service area number seven (7) of the star rating assessment (SRA) tools, namely client focus.“ without explaining the SRA tools. Therefore, the reader is not able to follow the context.

We have added a description on the SRA tool to help readers get a link between SRA tool, a star award and area 7 which is client focus.

It is not clear why the SRA dataset of 2017/18 was used. Why was there no newer data?

SRA dataset of 2017/18 is the newest nation-wide dataset so far. We have just started the third phase of SRA this year (2021/2022) by reaching out 10 among 26 regions available in the country. This is very expensive task and we are not sure yet if we will complete the task to all regions. The first SRA was conducted in 2015/16, however, it was not possible to maintain a quality database by that time.

Methods

In the Conceptual Framework the details of the content and implementation of the client service charter should be described. Is there a SOP which describes how to implement the CSC? How is it measured if a CSC is implemented or not?

As a country we have a guideline titled “National Client’s Service Charter for Health Facilities” in which chapter 4 describes on how to monitor and evaluate how facilities perform on implementing CSC.

Monitoring is conducted on quarterly basis through customer satisfaction surveys. The same tool that was used to collect data for this study is used for the surveys.

„due to one reason or another, facilities whose results were not found in the database were excluded from the analysis.“ => These reasons have to be explained in detail. Otherwise you have a sample with unknown selection criteria which influence your results.

The statement has been modified to make it clearer, thank you so much for the observation. The previous statement was not presenting the actual thing we did. We targeted all facilities that were assessed through Star Rating Assessment (SRA). However, some had missing data during data extraction and were excluded from the analysis.

Table 3: „Client service charter was implemented if all three indicators were met.“ Does this mean that all the displayed questions in Table 3 had to be answered with „Yes“ ? Please explain.
Thank you for the observation. To be more precise, the statement has been modified to *“The indicator for the client service charter was regarded implemented if all verification questions scored “Yes” for that particular indicator”*

The Analysis section has to be much more detailed. Statistical measures used have to be justified and described. The binary variable regarding patient satisfaction should be explained more clearly. Those who scored 8/10 were considered to be satisfied with the service?
More details have been added in different sub-sections of the Methodology section. Specifically, clarification on a binary variable has been made.

Results

There are 2766 (38%) PHC facilities not included in the analyses. A missing analysis regarding their characteristics has to be performed in order to demonstrate if you have a selective sample or not.

A sensitivity analysis was done and realized that excluded data had similar characteristics to those that were included. To clarify more, we have included a table numbered 4 that describe the characteristics of missing facilities.

Table 4: (currently Table 5)

As far as I have understood the authors have performed single-variable regressions first with each of the listed predictors and then included them all in one multivariate analyses. Please explain more precisely. Please give an indicator of model performance, e.g. McFadden´s R-square.
Thank you for the nice comment. However, there is a mixing understanding of the rationale for reporting indicators of model performance. For example, Giselmar et al ([https://doi.org/10.1177/0049124116638107](https://doi.org/10.1177%2F0049124116638107) ) in their study explain the following *“the McFadden values are not appropriate in cases with large sample sizes (n > 200) and/or strongly asymmetric distribution of observations to categories (ν > 1.6)”*

Therefore, we opted to include not the values to avoid potential confusion. However, we are ready to comply with the reviewer’s comment if he/she insists.

Discussion

Important points were raised for the future of the PHC system in Tanzania. Please explain further the discrepancy between the relatively low implementation of client service charter and the relatively high satisfaction of patients.

Thank you for the comment. We have added explaination in discussion and third paragraph of conclusion section to explain further the discrepancy between the relatively low implementation of client service charter and the relatively high satisfaction of patients.

Reviewer #4: The logical comments are as below.
1. The authors defined "Three clients were randomly selected at each dispensary and five at health centers and hospital level 1" as the facilities clients was satisfied with, and potential factors were examined for the associations with the client-satisfying facility. Three or five clients are not enough to evaluate the facilities in terms of client satisfaction. The authors should analyze the associations between client satisfaction and factors, not between facilities evaluated by a limited number of clients and factors.

We agree with the reviewer; three to five clients are few to evaluate client satisfaction at the facility. However, However, the experience during data collection showed the number of clients attending the majority of facilities i.e. dispensaries was very low to the extent it was difficult in some instances to achieve the required number of interviewees. The findings of this study are presented as “satisfaction on a day of the visit” and should be translated with such a precaution. We have included this as one of the limitations of the study in the manuscript text.

2. The authors did not describe the method of random selection of clients. The readers cannot judge whether the clients were sampled randomly or not.

We have added a description (in manuscript text) on how clients for exit interviews were randomly selected in the current version.

“If there was more than one client exiting the facility, the first client to be interviewed was randomly selected by using a lottery system. In this system, each client was assigned a number and thereafter each number was written on each piece of paper. All papers were shuffled well and one paper was picked randomly to obtain the first interviewee. After returning from an interview, the data collector selected the next patient entering the consultation room until the targeted sample size was achieved.”

3. The characteristics of clients were not described. The readers cannot understand whose evaluation was the base of satisfaction.

This is one of the limitations of the study, we have it in the manuscript text. The database we used does not contain the characteristics of the clients interviewed.

4. The characteristics and qualification of interviewers were not described.

We have added a special sub-section named “data sources” under “Study variables and collection of data”. In the second paragraph (with the reference), we have described the characteristics and quality of data collectors.

5. Figure 2 is hard to be interpreted. The title says "Proportion of primary health facilities whose clients are satisfied". For example, what did the author mean for the proportion of "Charter displayed?"?
The figure has been revised both structurally and descriptive to make it clearer. Thank you for the comment.

The technical comments are as follows.
1. The abbreviation must be defined at the first appearance with the full spelling, and the abbreviation should be used thereafter. For example, "CSC".

The document has been reviewed and corrections made as per reviewer’s comment.

2. In Table 2, delete "=" after "Wait less than 60 minutes".

The symbol has been omitted, thank you for the observation.

3. In Table 4, COR of 1.125 should be replaced with "1.13". The term "Ref" is dropped at four parts.

Thank you for the comment, changes have been made.

**Reviewer #5: Dear authors**
I would like to thank you for giving me the opportunity to review this well-written manuscript entitled “Clients satisfaction at primary health-care facilities and its association to the status of client service charter implementation in Tanzania”. The main objective of the study was to determine clients’ satisfaction at PHC facilities and its association with the status of client service charter implementation in Tanzania. This work is well designed and well well-written script that can improve the delivering PHC services. Therefore, I think the manuscript can be considered seriously for publishing. I have just one comment:
1. I would like to see some information regarding Tanzania such as population, socio-economic status, and PHC in the introduction.
Best wishes

I thank the reviewer for the positive comments. The recommendations have been incorporated in the introduction section.
